# Widening social inequality in life expectancy in Denmark. A register-based study on social composition and mortality trends for the Danish population

**DOI:** 10.1186/1471-2458-12-994

**Published:** 2012-11-17

**Authors:** Henrik Brønnum-Hansen, Mikkel Baadsgaard

**Affiliations:** 1 Department of Public Health, University of Copenhagen, Øster Farimagsgade 5, 1014, Copenhagen, Denmark; 2 Economic Council of the Labour Movement, Reventlowsgade 14, 1651, Copenhagen, Denmark

**Keywords:** Education, Income, Life expectancy, Social inequality, Trends

## Abstract

**Background:**

Dynamics of the social composition of the population might influence the interpretation of statements of the increasing gap of social inequality in life expectancy. The aim of the study was to estimate trends during a quarter of a century in social inequality in life expectancy and to compare results based on different social stratifications.

**Methods:**

Life tables by sex and various levels of education and income were constructed for each year in the period 1987–2011 by linking individual data from nationwide registers comprising information on all Danish citizens on date of birth, date of death, education and income. Trends in life expectancies were compared for different categories of social grouping of the population.

**Results:**

When categories of educational level were kept fixed, implying a decreasing proportion of persons with a short education, the educational inequality in life expectancy increased. Thus, the difference in life expectancy at age 30 between men with primary or lower secondary education and men with tertiary education increased from 4.8 years in 1987 to 6.4 years in 2011. For women the difference increased from 3.7 years in 1987 to 4.7 in 2011. A similar growing social disparity was observed when educational level was based on quartiles established from prescribed length of education. A considerable increasing inequality was reached for men when the population was divided in quartiles of equivalent disposable income, whereas the change was only modest for women.

**Conclusions:**

During the past 25 years, the social gap in life expectancy has widened in Denmark. This conclusion could not be explained by changes of the social compositions of the population.

## Background

Since 1970 Statistics Denmark has monitored social differences in mortality among Danes on the basis of nationwide registration of social position and mortality. The overall observation during the 1990s was a marked social inequality in mortality and a tendency to a widening gap between educational groups [1]. In Denmark as in other Nordic countries and populations deaths from cardiovascular diseases contribute substantially to a growing social inequality in mortality [2,3].

When social prosperity grows to the benefit of the broad population public health improves, but not necessarily with the same speed for all social categories. If no gain in prosperity is obtained among the least favoured social groups the social gap in health status is likely to be widened. Societal dynamics change the social composition of the population implying modifications of characteristics of social categories and bring up how to understand social inequality trends in morbidity and mortality.

**Figure 1:**
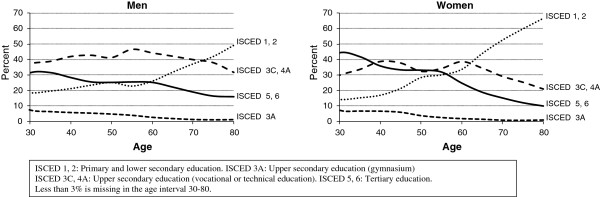
Educational levels by age and sex, Denmark 2010.

Recently we reported that the social gap in life expectancy and health expectancy has widened in Denmark [4,5]. In these studies social categories were kept fixed on the basis of three levels of education. However, the population educational level has improved and the size of the educational subpopulations has changed. For instance, the share of women with a low educational level (i.e. primary and lower secondary school) declined from 51% in 1987 to 23% in 2011 in the age bracket 30–64 years. Figure 1 shows that the educational transition is particularly considerable among women. More education is offered to young people today than previously and as a consequence of the general raise of educational level people with lesser education represent a diminishing and probably increasingly disadvantaged group whereas the proportion of people with more education grows and “attenuates” their advantageous position. Therefore, to better understand differential secular trends in mortality account should be taken of dynamics of the social composition of the population. The importance of this aspect and the interpretation of changes in social inequalities in health has been discussed in several studies and illustrated previously [6-8].

A unique personal identification number assigned to all Danish citizens make it possible to link various register data at the individual level such as successive information on education, income and vital status. This permits flexibility to defining and characterizing various population groups and to study differential mortality in the population. The purpose of the study was to estimate trends during a quarter of a century in social inequality in life expectancy and take into account that the characteristics of social categories and social composition of the population has changed.

## Methods

Statistics Denmark collects systematically educational data from the Ministry of Education and data on income from the Danish Tax and Customs Administration. Information for all Danish citizens is updated annually. However, the organizing of the systematic data collection on education started with an age limit restriction meaning that information on education during our study period 1987–2011 was only available for people under 65. At the end of the period data was available up to the age of 88 and we applied the relative disparities in death rates in 2010 between educational groups for ages above 65 for the entire period. Educational-group-specific life expectancy was estimated at age 30. In order to handle the upper age limit restriction on education information we also calculated trends in partial life expectancy, i.e. expected lifetime between the ages of 30 and 65. Equivalent disposable income was the basis for investigating income differentials in life expectancy for a new-borne.

For each year between 1987 and 2011 we calculated sex- and age-specific death rates for various educational and income groups by linking register data on vital status with that on education and income, respectively. Sex-specific life tables were constructed for each calendar year and grouped as to educational or income level. Age-group-specific contributions to changes in life expectancy were estimated by decomposition [9].

We used two classifications of education:

1. Four fixed levels: Persons with primary and lower secondary education (ISCED 1, 2), persons with upper secondary education (gymnasium) (ISCED 3A), persons with upper secondary education (vocational or technical education) (ISCED 3C, 4A), persons with tertiary education (ISCED 5, 6).

2. Educational quartiles: Prescribed length of education in months, from which the social grouping was established by dividing men and women in quartiles at any age and calendar year. Length of schooling/studying is unevenly distributed and persons with length of education at the 25%, 50% and 75% of the cumulative frequency curve were randomly assigned to one of the two relevant neighbouring quartiles.

Equivalent disposable income takes into account that consumption possibilities vary between households due to disparity in size and constitution [10]. Individual income was based on equivalent disposable income and the population was divided into four quartiles analogous to the grouping in educational quartiles. Results are shown for current income, but with a view to take into account that potentially lethal disease may cause income reduction we also computed life expectancy using income 1, 3 and 5 years previously.

## Results

**Figure 2:**
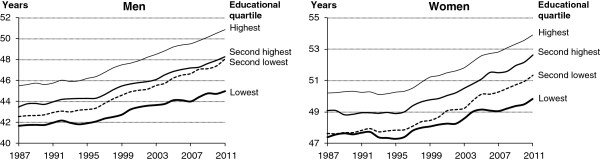
Trends in life expectancy at age 30 by educational quartiles, Denmark 1987–2011.

Life expectancy for 30-year-old men with primary and lower secondary education increased by 7.7% from 41.7 years in 1987 to 44.9 years in 2011 (Table 1). For men with tertiary education the increase in life expectancy at age 30 was 10.3% from 46.5 years in 1987 to 51.3 years in 2011. Thus, the difference in life expectancy between men with tertiary education and men with primary and lower secondary education increased by 1.6 years from 4.8 years to 6.4 years (Table 1). During the last 25 years life expectancy grew less for women than for men but a similar pattern of increasing inequality between educational groups was observed. Life expectancy for 30-year-old women with primary and lower secondary education increased by 4.9% from 47.4 years in 1987 to 49.7 years in 2011, whereas the increase among women with tertiary education was 6.5% and the difference in life expectancy between the highest and lowest educational levels increased by 1.0 year from 3.7 years to 4.7 years (Table 1). Comparison between educational quartiles did not change the overall pattern. But the social differences are less pronounced but tend to grow more (Table 1). The difference between the two lowest educational quartiles was relatively small in 1987 – particularly among women. This is due to the high prevalence of people with equally low educational level resulting in a high proportion of persons who were randomly allocated to the two lowest quartiles. This mechanism moves forward during the study period and increases the proportion of random allocation between the two middle quartiles and particularly so for the younger ages. Figure 2 shows trends in life expectancies for educational quartiles.

**Table 1:** Life expectancy at age 30 by four fixed educational levels and by educational quartiles, 1987 and 2011

	**Men**			**Women**		
	**Life expectancy**			**Life expectancy**		
	**1987 (years)**	**2011 (years)**	**Difference (years)**	**1987 (years)**	**2011 (years)**	**Difference (years)**
Fixed educational level (ISCED)						
Tertiary education (5, 6)	46.5	51.3	4.8	51.1	54.4	3.3
Upper secondary (vocational or technical) education (3C, 4A)	43.5	48.3	4.8	49.7	52.8	3.1
Upper secondary (gymnasium) education (3A)	44.6	49.0	4.4	49.4	52.6	3.2
Primary and lower secondary education (1, 2)	41.7	44.9	3.2	47.4	49.7	2.3
Difference between highest and lowest educational level	4.8	6.4	1.6	3.7	4.7	1.0
Educational quartile						
Highest educational group	45.5	50.8	5.3	50.2	53.9	3.7
Second highest educational group	43.4	48.3	4.9	49.1	52.6	3.5
Second lowest educational group	42.6	48.0	5.4	47.6	51.3	3.7
Lowest educational group	41.7	45.0	3.3	47.4	49.8	2.4
Difference between highest and lowest educational quartile	3.8	5.8	2.0	2.8	4.1	1.3

**Figure 3:**
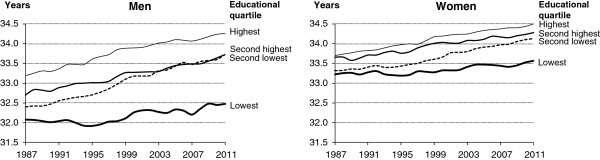
Trends in partial life expectancy (age 30 – 65) by educational quartiles, Denmark 1987–2011.

A widening social inequality in expected lifetime between the ages of 30 and 65 also appears from Figure 3. The gap in partial life expectancy between the lowest and the highest educational quartile increased by 0.66 years for men and 0.45 years for women. For both sexes the two middle quartiles converged. Thus, in 1987 the partial life expectancy differed by 0.31 years for men and 0.34 years for women. In 2011 the difference vanished for men and was only 0.15 years for women. As stated above, the explanation is the increasing proportion of the random allocation between the middle quartiles.

**Figure 4:**
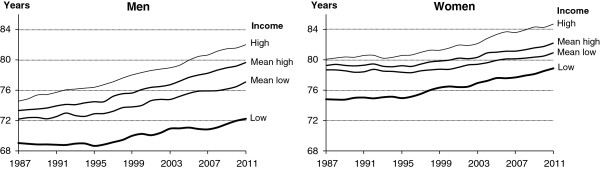
Life expectancy at age 0 by sex and equivalent disposable income quartiles, Denmark 1987–2011.

Figure 4 shows trends in life expectancy at age 0 for the four equivalent disposable income quartiles during the study period and Table 2 presents results for 1987 and 2011. For men in the lowest quartile expected lifetime was 69.1 years in 1987 and 72.2 years in 2011 – an increase of 4.6%. For men in the highest income quartile life expectancy increased by 9.9% (from 74.6 years in 1987 to 82.0 years in 2011). Also for women the social inequality is substantial, but the change was less marked. In particular, women in the lowest income quartile gained more in life expectancy than women in the middle quartiles (Table 2). Thus, the difference in life expectancy between women in the high and low income categories rose only by 0.5 years (from 5.3 years in 1987 to 5.8 years in 2011). The results were robust with regard to whether income was chosen to be current or lagged. However, the gap reduces when lagged income was used, but the widening inequality during the period remained.

**Table 2:** Life expectancy at age 0 by equivalent disposable income quartiles, 1987 and 2011

	**Men**			**Women**		
	**Life expectancy**			**Life expectancy**		
**Income quartile**	**1987 (years)**	**2011 (years)**	**Difference (years)**	**1987 (years)**	**2011 (years)**	**Difference (years)**
High income	74.6	82.0	7.4	80.1	84.7	4.6
High mean income	73.3	79.6	6.3	79.2	82.2	3.0
Low mean income	72.2	77.1	4.9	78.6	80.9	2.3
Low income	69.1	72.2	3.2	74.8	78.9	4.1
Difference between high and low income	5.5	9.8	4.2	5.3	5.8	0.5

Further investigations of changed mortality disclosed that the death rates for men in the age groups 50–59 and 60–69 did not develop as favourable in the lowest income quartile as in the highest income quartile (Table 3). Among the youngest age groups the lowest income quartile made better reflecting a higher proportion of students (with low income) in 2011 than in 1987. An obvious explanation of the gender difference among the elderly is that almost no elderly women smoked in 1987 and that well off women were the first to take up the habit after the Second World War. A similar decomposition of life expectancy for the educational quartiles showed the same pattern of increasing difference between the highest and the lowest quartile by age (for the age groups 30–39, 40–49 and 50–59). Obviously, for ages above 60 the differences were almost eliminated because of the assumption of constant relative disparities between educational groups for ages above 65.

**Table 3:** Age specific contributions to changes between 1987 and 2011 in life expectancy in the highest and lowest quartile of equivalent disposable income

	**Age group**							
**Income quartile**	**0-19**	**20-29**	**30-39**	**40-49**	**50-59**	**60-69**	**70+**	**Total**
Men								
High income	0.3	0.3	0.5	0.8	1.4	2.1	2.0	7.4
Low income	0.5	0.4	0.3	0.0	−0.2	0.6	1.6	3.2
Difference between high and low income	−0.3	−0.1	0.2	0.8	1.6	1.6	0.4	4.2
Women								
High income	0.2	0.1	0.3	0.5	1.1	1.3	1.1	4.6
Low income	0.4	0.2	0.2	0.2	0.1	0.4	2.7	4.1
Difference between high and low income	−0.2	−0.1	0.1	0.3	1.0	0.9	−1.6	0.5

## Discussion

During a long period before the mid 1990s life expectancy did not change much in Denmark [11]. The stagnation in life expectancy among persons with a low educational level or income appears clearly from the figures. At the end of the century life expectancy began to increase for all groups although with a slower rate among the socially disadvantaged groups.

The overall conclusions were remarkably consistent transversely to the different methods. We expected that the considerably changes in the size of the educational subpopulations during a quarter of a century would result in different trends in social inequality depending on whether the four educational levels were defined by fixed categories or by “flowing” quartiles. One result leaps to the eye when trends are estimated on the basis of equivalent disposable income. Life expectancy among women in the lowest income quartile improved more than it did for women who belong to the two middle quartiles. It appears from Figure 4 that the middle-income half of the Danish female population lag behind, but a similar trend was not seen for educational differentials (Figure 2). This paradox might be related to changes in the labour market structure, the flexicurity model, and wage rate developments, family constellations and how these factors interact with the construct of equivalent disposal income.

No simple theory exists that explain the different developments, but to some extent mortality trends are related to health-related behaviour and the so-called “intervention generated inequalities” that characterizes the Scandinavian welfare societies might contribute to increasing social inequality in health [12,13].

Health-related behaviours vary between social groups. For instance smoking is more prevalent among social disadvantaged people. But the social gradient in life expectancy is seen irrespectively of smoking category [14]. The difference between 30-year-old male never smokers with a high and a low educational level was 2.3 years, whereas the similar difference among heavy smokers was 3.7 years. The educational disparities for women were 1.3 years for never smokers and 1.9 years for heavy smokers [14]. Thus, the impact of smoking seems to be greater among people with a low educational level in spite of a shorter lifetime. This might in part be explained by higher tobacco consumption among heavy smokers or more clustering of risk factor exposures among lower educated smokers.

The decline in smoking prevalence is faster among socially advantaged than socially disadvantaged people [15]. Thus, heavy smoking prevalence among men with less than 10 years of education increased from 26.0% in 1987 to 31.4% in 2005 whereas it decreased from 12.2% to 8.8% among men with more than 14 years of education. For women with less than 10 years of education the prevalence increased from 18.5% to 29.1% and for high educated women it decreased from 17.6% to 7.6%. The same direction of trends were seen in all age groups with the only exception that prevalence of heavy smoking among high educated men aged 65 and over increased from 5.2% in 1987 to 7.7% in 2005 [15]. Prevalence of moderate smoking declined in all groups.

High alcohol consumption is characterized by a general upward trend, although stagnation among the highest educated persons results in almost equality between educational groups [15]. Overweight and obesity increased in all educational groups, but the strong social gradient is maintained [15].

A result based on data from the British Whitehall II study suggests that health-related behaviours (smoking, alcohol consumption, unhealthy diet and physical inactivity) assessed longitudinally could explain 72% of social inequalities in mortality [16].

A recent Finnish study investigating trends in income differentials in life expectancy for 35 year-olds found that the difference in life expectancy between the lowest and the highest income quintile widened by 5.1 years for men and 2.9 years for women from 1988 to 2007, the difference being 12.5 years for men and 6.8 years for women in 2007 [17]. For both sexes the main reason for the increased disparity was stagnation among those with the lowest income. For men this is in line with our results, whereas the change was more favourable among Danish women in the lowest income quartile. However, social classification based on educational level in our study also indicated poor improvement in survival among women with the shortest education.

The difference in life expectancy between higher non-manual and unskilled Swedish 20-year-old men increased from 2.1 years in 1980 to 3.8 years in 1997. For Swedish women the increase was from 1.6 years to 2.2 years [18].

Also in Belgium life expectancy increases more among people with a high educational level compared with people with a low educational level and for women without formal education life expectancy even seems to decrease [19].

Studies in the USA similarly demonstrate increasing social inequality in life expectancy [20-22]. Thus, only modest improvement in life expectancy during 1981–2000 was seen among less educated black women and white non-Hispanics men and women, while the more educated groups experienced substantial gains [21]. Another US study estimated trends in life expectancy differentials by lifetime earnings and found that between 1990 and 2000 differentials in life expectancy between ages 35 and 76 has increased from 2.7 years to 3.6 years between men in the lowest and the highest lifetime earning quintile. For women the difference increased from 0.7 years to 1.5 years [22].

An important strength of our study was that death rates were based on data for all Danish citizens although information on educational level was only available for ages under 65 for the entire period. As sex- and age-specific death rates were calculated exactly within each educational and income level, life tables could be constructed, apart from educational-specific figures for the elderly. However, the lack of educational information on the elderly is due to the systematic data collection procedure at Statistics Denmark and not related to social characteristics. We assumed that the relative disparities in death rates in 2010 between educational groups were valid for ages above 65 for the entire period. If we made the conservative assumption that the mortality rates after age 65 were equal for all educational groups the difference between the lowest and the highest educational quartile in life expectancy at age 30 grew from 2.0 years in 1987 to 4.1 years in 2011 for men and from 1.2 years to 2.6 years for women.

The division into educational and income quartiles defines synthetic life tables because educational or income membership of quartiles changes during life, which is not reflected in the period life table birth cohorts. But this construct makes comparisons between social categories possible and is similar to the classical life table assumption of constant age specific death rates for all birth cohorts.

## Conclusion

The gap between favoured and less favoured people in life expectancy has widened during a quarter of a century. The conclusion could not be explained by changes of the social composition of the population.

Differences in health-related behaviours are parts of the explanation for the social inequality in mortality and it is a continuous challenge to reduce unhealthy lifestyle by health promotion targeting high-risk subpopulations, including socially disadvantaged groups. The labour market seems to be more competitive and the conditions more ruthless, which might have placed a greater strain on socially disadvantaged people than on socially advantaged people and the impact of the financial crises might elevate the social inequality in health status and mortality. If reduction of social inequality is on the political agenda, reforms of living conditions and social welfare for the benefit of the least socially favoured groups are needed to improve housing standards, eliminate stressors and other factors injurious to health in the local environment, reduce high workloads and improve job security.

## Competing interest

The authors declare that they have no competing interests.

## Authors’ contributions

HBH and MB initiated the study. MB undertook the analysis. HBH wrote the manuscript and both authors have seen and approved the final version.

## Pre-publication history

The pre-publication history for this paper can be accessed here:

http://www.biomedcentral.com/1471-2458/12/994/prepub
